# Supplementary material for: Altered Gut Microbiota and Its Clinical Relevance in Mild Cognitive Impairment and Alzheimer’s Disease: Shanghai Aging Study and Shanghai Memory Study
Source: Nutrients. 2022 Sep 23;14(19):3959. doi: 10.3390/nu14193959 (PMC9570603; doi:10.3390/nu14193959)
Supplement: Supplementary file 1 [file nutrients-14-03959-s001.zip › nutrients-1858643-supplementary.pdf]

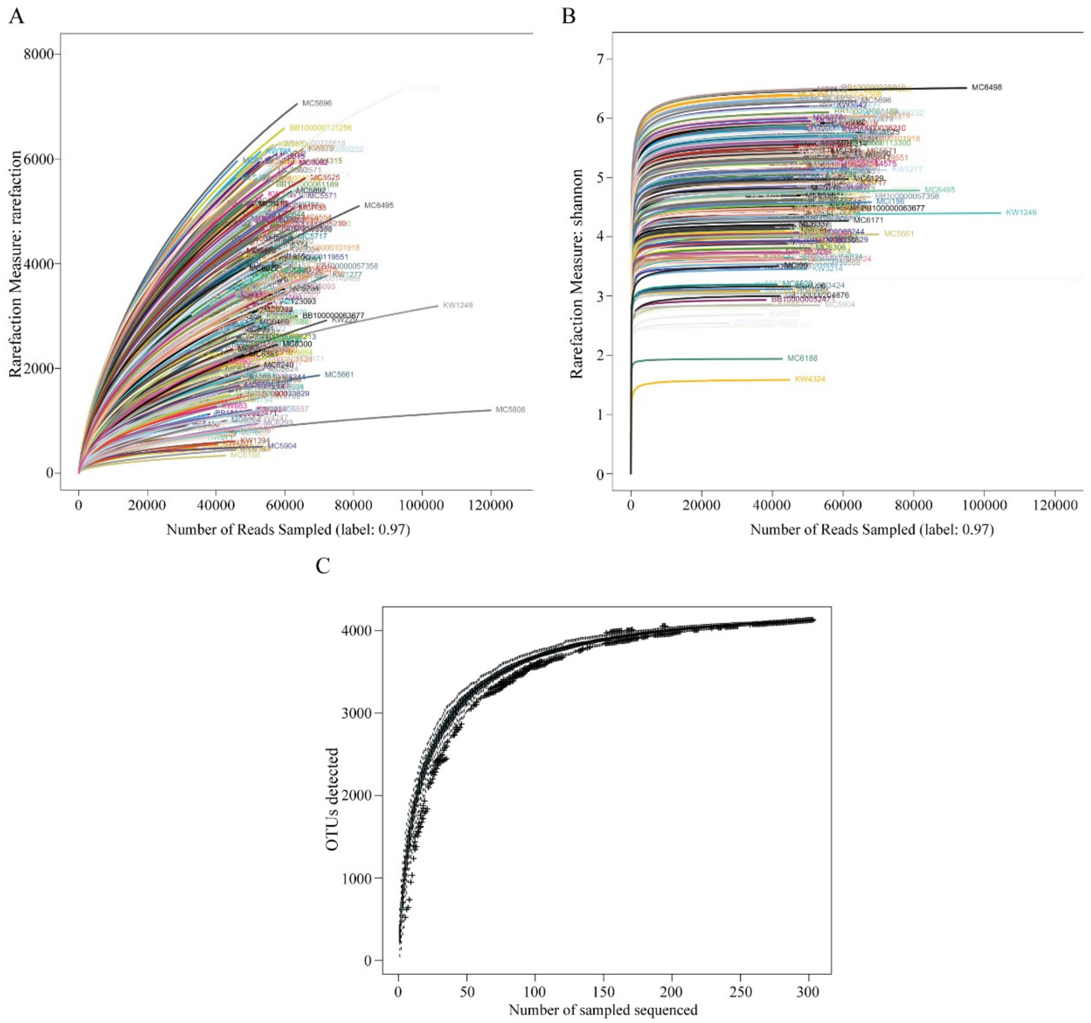

Figure S1. Alpha diversity analysis of the gut microbiota in NC, MCI, and AD.

Alpha diversity analysis of the gut microbiota among NC, MCI, and AD, illustrated by A. Rarefaction curves; B. Shannon-Wiener curves; C. Species accumulation curves.

NC, cognitively normal controls; MCI, mild cognitive impairment; AD, Alzheimer's disease; OTUs, Operational taxonomic units.

Table S1. The correlation (Corr) coefficients and P value of the five specific gut microbiota taxa with clinical characteristics

	Erysipelatoclostridiaceae	Erysipelotrichales	Saccharimonadales	Patescibacteria	Saccharimonadia
Age	0.066024202	0.048186319	0.162941006	0.162941006	0.162941006
	P＞0.05	P＞0.05	P=0.0045	P=0.0045	P=0.0045
Education	-0.04574294	-0.035431348	-0.101498002	-0.101498002	-0.101498002
	P＞0.05	P＞0.05	P＞0.05	P＞0.05	P＞0.05
MMSE	-0.079646069	-0.092068593	-0.285270844	-0.285270844	-0.285270844
	P＞0.05	P＞0.05	P=4.61e-07	P=4.61e-07	P=4.61e-07
MoCA	-0.105802869	-0.113735467	-0.228162636	-0.228162636	-0.228162636
	P＞0.05	P＞0.05	P=5.31e-04	P=5.31e-04	P=5.31e-04
Z_memory	-0.160678551	-0.18852155	-0.174634342	-0.174634342	-0.174634342
	P=0.0087	P=0.002	P=0.0043	P=0.0043	P=0.0043
Z_attention	-0.089689796	-0.115544157	-0.057319084	-0.057319084	-0.057319084
	P＞0.05	P＞0.05	P＞0.05	P＞0.05	P＞0.05
Z_visuospatial	-0.12996039	-0.116307259	-0.105414672	-0.105414672	-0.105414672
	P=0.0341	P＞0.05	P＞0.05	P＞0.05	P＞0.05
Z_executive	-0.082433706	-0.075605569	-0.209663066	-0.209663066	-0.209663066
	P＞0.05	P＞0.05	P=5.78e-04	P=5.78e-04	P=5.78e-04
Z_language	-0.094812701	-0.112472813	-0.189420894	-0.189420894	-0.189420894
	P＞0.05	P＞0.05	P=0.0019	P=0.0019	P=0.0019
CDR	0.113356807	0.127017794	0.314628499	0.314628499	0.314628499
	P=0.0494	P=0.0276	P=2.43e-08	P=2.43e-08	P=2.43e-08
ADL	-0.023100965	-0.008717582	0.231340891	0.231340891	0.231340891
	P＞0.05	P＞0.05	P=5.87e-05	P=5.87e-05	P=5.87e-05

MMSE, Mini-mental State Examination; MoCA, Montreal Cognitive Assessment; ADL, Activities of Daily Living; CDR, Clinical Dementia Rating. The composite Z scores were computed for specific cognitive domains including memory, attention, visuospatial ability, Language, and Executive function.

Table S2. The P value of abundance comparisons of five specific taxa among different clinical subgroups

Subgroup comparison	Erysipelatoclostridiaceae	Erysipelotrichales	Saccharimonadales	Patescibacteria	Saccharimonadia
NC *vs.* MCI	0.005645	0.02662	0.007575	0.007575	0.007575
NC *vs.* AD	6.32E-06	8.28E-06	8.37E-05	8.37E-05	8.37E-05
MCI *vs.* AD	0.0299	0.008745	0.04777	0.04777	0.04777
Comparison among NC, MCI and AD	3.03E-05	4.54E-05	0.000211	0.000211	0.000211
CDR 0 *vs.* 0.5	3.10E-05	0.0003111	0.004855	0.004855	0.004855
CDR 0 *vs.* 1	7.57E-05	2.47E-05	0.00652	0.00652	0.00652
CDR 0 *vs.* 2	6.13E-06	4.59E-05	0.000779	0.000779	0.000779
CDR 0 *vs.* 3	0.0286	0.07643	0.01093	0.01093	0.01093
CDR 0.5 *vs.* 1	0.3456	0.05666	0.4117	0.4117	0.4117
CDR 0.5 *vs.* 2	0.0408	0.06467	0.07114	0.07114	0.07114
CDR 0.5 *vs.* 3	0.5797	0.4941	0.03867	0.03867	0.03867
CDR 1 *vs.* 2	0.3037	0.8813	0.4025	0.4025	0.4025
CDR 1 *vs.* 3	0.921	0.9125	0.1355	0.1355	0.1355
CDR 2 *vs.* 3	0.7158	0.8555	0.2676	0.2676	0.2676
Comparison among CDR 0, 0.5, 1, 2 and 3	2.52E-06	1.19E-05	0.0005255	0.0005255	0.0005255
APOE 4- *vs.* *APOE* 4+	0.01978	0.004703	0.01303	0.01303	0.01303

NC, cognitively normal controls; MCI, mild cognitive impairment; AD, Alzheimer's disease. AD, Alzheimer's disease; CDR, Clinical Dementia Rating. APOE, apolipoprotein E.

Table S3. The P value of abundance comparisons of five specific taxa among different clinical subgroups in participants aged ≥60yrs.

Subgroup comparison	Erysipelatoclostridiaceae	Erysipelotrichales	Saccharimonadales	Patescibacteria	Saccharimonadia
NC vs. MCI	0.006	0.035	0.031	0.031	0.031
NC vs. AD	< 0.001	< 0.001	< 0.001	< 0.001	< 0.001
MCI vs. AD	0.202	0.064	0.195	0.195	0.195
Comparison among NC, MCI and AD	< 0.001	< 0.001	< 0.001	< 0.001	< 0.001
CDR 0 vs. 0.5	0.001	0.008	0.077	0.077	0.077
CDR 0 vs. 1	0.001	< 0.001	0.092	0.092	0.092
CDR 0 vs. 2	0	< 0.001	0.003	0.003	0.003
CDR 0 vs. 3	1	1	0.095	0.095	0.095
CDR 0.5 vs. 1	1	0.713	1	1	1
CDR 0.5 vs. 2	0.427	0.622	0.565	0.565	0.565
CDR 0.5 vs. 3	1	1	0.725	0.725	0.725
CDR 1 vs. 2	1	1	1	1	1
CDR 1 vs. 3	1	1	1	1	1
CDR 2 vs. 3	1	1	1	1	1
Comparison among CDR 0, 0.5, 1, 2 and 3	< 0.001	< 0.001	0.001	< 0.001	< 0.001
APOE 4- vs. APOE 4+	< 0.001	< 0.001	0.012	0.012	0.012

NC, cognitively normal controls; MCI, mild cognitive impairment; AD, Alzheimer's disease. AD, Alzheimer's disease; CDR, Clinical Dementia Rating. APOE, apolipoprotein E.
